# Immobilization of GH78 ⓹-L-Rhamnosidase from *Thermotoga petrophilea* with High-Temperature-Resistant Magnetic Particles Fe_3_O_4_-SiO_2_-NH_2_-Cellu-ZIF8 and Its Application in the Production of Prunin Form Naringin

**DOI:** 10.4014/jmb.2004.04055

**Published:** 2020-06-24

**Authors:** Jin Xu, Xuejia Shi, Xiaomeng Zhang, Zhenzhong Wang, Wei Xiao, Linguo Zhao

**Affiliations:** 1 Jiangsu Co-Innovation Center of Efficient Processing and Utilization of Forest Resources, Nanjing Forestry University, 159 Long Pan Road, Nanjing 210037, P.R. China; 2 College of Chemical Engineering, Nanjing Forestry University, 159 Long Pan Road, Nanjing 210037, P.R. China; 3 Jiangsu Kanion Pharmaceutical Co., Ltd., 58 Haichang South Road, Lianyungang 222001, Jiangsu Province, P.R. China

**Keywords:** Immobilization, magnetic microspheres, alpha-L-rhamnosidase, naringin, reusability

## Abstract

To efficiently recycle GH78 thermostable rhamnosidase (TpeRha) and easily separate it from the reaction mixture and furtherly improve the enzyme properties, the magnetic particle Fe_3_O_4_-SiO_2_-NH_2_-Cellu-ZIF8 (FSNcZ8) was prepared by modifying Fe_3_O_4_-NH_2_ with tetraethyl silicate (TEOS), microcrystalline cellulose and zinc nitrate hexahydrate. FSNcZ8 displayed better magnetic stability and higher-temperature stability than unmodified Fe_3_O_4_-NH_2_ (FN), and it was used to adsorb and immobilize TpeRha from *Thermotoga petrophilea* 13995. As for properties, FSNcZ8-TpeRha showed optimal reaction temperature and pH of 90°C and 5.0, while its highest activity approached 714 U/g. In addition, FSNcZ8-TpeRha had better higher-temperature stability than FN. After incubation at 80°C for 3 h, the residual enzyme activities of FSNcZ8-TpeRha, FN-TpeRha and free enzyme were 93.5%, 63.32%, and 62.77%, respectively. The organic solvent tolerance and the monosaccharides tolerance of FSNcZ8-TpeRha, compared with free TpeRha, were greatly improved. Using naringin (1 mmol/l) as the substrate, the optimal conversion conditions were as follows: FSNcZ8-TpeRha concentration was 6 U/ml; induction temperature was 80°C; the pH was 5.5; induction time was 30 min, and the yield of products was the same as free enzyme. After repeating the reaction 10 times, the conversion of naringin remained above 80%, showing great improvement of the catalytic efficiency and repeated utilization of the immobilized α-L-rhamnosidase.

## Introduction

α-L-Rhamnosidase (E.C. 3.2.1.40) cleaves terminal α-L-rhamnosidase bonds specifically in a large number of natural products [1, 2]. The enzyme is widespread in nature and is reported from animal tissues, plants, yeasts, fungi and bacteria [3]. It is a biotechnologically important enzyme due to its applications in debittering and clearance of citrus fruit juices, enhancement of wine aromas and derhamnosylation of many natural products containing terminal α-L-rhamnose into compounds of pharmaceutical interest [4]. For example, α-L-rhamnosidase from *Bifidobacterium breve* can increase the yield of ginsenoside Rg1 in *Panax ginseng* [5], and also promote the biotransformation of rutin to isoquercitrin [6]. α-L-Rhamnosidases from *Aspergillus terreus* and *Pichia pastoris Mut(S)* strain, the current most preferred microorganisms for recombinant enzyme production owing to their efficient expression systems, can transform rutin into isoquercitrin [7, 8]. There are many reports on α-L-rhamnosidase, but few about thermostable α-L-rhamnosidase. Although it boasts high specificity in the biocatalytic transformation of glycosides than other enzymes [9], it may encounter the same problems, such as low expression and high cost.

GH78 thermostable rhamnosidase (E.C. 3.2.1.40) (TpeRha) from *Thermotoga petrophila* 13995 is a high-temperature-resistant enzyme. It similarly encounters the problem of low expression, as mentioned above, but has several advantages, including enzymatic specificity in the transformation of naringin to prunin and high-temperature enzymatic efficiency over normal-temperature rhamnosidases. Moreover, the positive effects of high-temperature reaction systems include higher substrate solubility, such as low water-soluble flavonoid and saponin components, simple separation of the later products, and termination of the addition of solubilizing agents, such as methanol, ethanol, and DMSO [4].

Immobilization is one of the commonest technical means to obtain efficient biocatalysts using a solid support. Immobilization techniques could allow repeated and continuous operation of enzymes and improve enzyme properties. Fe_3_O_4_ nanoparticles can be used as support carriers for immobilized enzyme owing to their magnetic force and metal-organic frameworks (MOFs) [10]. The magnetic property of Fe_3_O_4_ nanoparticles leads to their easy separation from the reaction mixture and high reusability of biocatalysts [11]. Recent studies show that this magnetic force could be strengthened by modifying Fe_3_O_4_ nanoparticles with functional groups [12]. Moreover, they have been equipped with catalytic, biotechnological, and biomedical devices in the mixture. For example, grapheme oxide is integrated with Fe_3_O_4_ nanoparticles, but the combined supporting material can be separated rapidly with an external magnetic field [13]. On the other hand, the surface of modified Fe_3_O_4_ nanoparticles could be enlarged and retained with functional groups. However, its applicability in immobilizing heat-resistant enzymes is limited by the barriers, easy acidification, and poor high-temperature stability of Fe_3_O_4_ [14, 15]. It is noteworthy that cellulose combined with prepared zeolitic imidazolate (ZIF-8) framework is considered a promising surface material [16, 17]. The hydroxyl groups of cellulose, which coats the MOFs, not only increase metal ion adsorption to enhance catalytic activities but also improve acidification, poor high-temperature stability, and organic solvent tolerance [18, 19]. Moreover, several studies showed that composite microparticle cores obtained through Fe_3_O_4_ nanomaterials functionalized with silica could significantly improve enzyme stability [20, 21].

Here, we aimed to examine the immobilization of TpeRha from *T. petrophila* 13995 using Fe_3_O_4_-SiO_2_-NH_2_-cellu-ZIF8. This modified MOF, compared with prepared Fe_3_O_4_ nanoparticles, had excellent high-temperature stability and acid resistance. The immobilized enzyme was applied to the transformation of naringin, which combined the advantages of immobilized enzyme and high-temperature enzyme, improved the enzymatic properties of high-temperature α-L-rhamnosidase, and enhanced the catalytic performance and reusability of the enzyme. The results provide technical support for the large-scale application of TpeRha in rhamnoid-containing natural products.

## Materials and Methods

### Materials

TpeRha from *T. petrophila* DSM 13995 was expressed by recombinant *Escherichia coli* BL21 (DE3) [22]. The materials used to prepare nano-Fe_3_O_4_ include FeCl_3_·6H_2_O, FeCl_2_·4H_2_O, ethanol, and 25% ammonia water purchased from Sinopharm Group Chemicals Co., Ltd. (China). The materials used for Fe_3_O_4_ surface modification include 98% 3-aminopropyltriethoxysilane (APTES) purchased from Sinopharm Group Chemicals Co., Ltd., tetraethyl silicate (TEOS) purchased from Macleans (China), 2-methylimidazole and naringin purchased from In Aladdin (China), and zinc nitrate hexahydrate purchased from Nanjing Chemical Reagent Co., Ltd. (China).

### Methods

**Enzyme Preparation Procedure**

The strain was inserted into TB medium at a 9:1 volume ratio of A:B as follows: A: 1% maltodextrin, 1.2%peptone and 2.4% yeast extract; B: 2.3 g of potassium dihydrogen phosphate and 16.43 g of potassium monohydrogen phosphate dissolved in 100 ml of distilled water. The mixture was cultured at 37°C to an OD_600_ of 0.6-0.8. After 48 h of induction at 37°C with isopropyl β-D-thiogalactoside (IPTG) at a final concentration of 0.01 mmol/l, the bacteria was collected. After ultrasonication, the free enzyme TpeRha (0.4 mg/ml) was obtained.

**Preparation of Nano-Fe_3_O_4_**

In this experiment, the chemical co-precipitation method was used to prepare nano-Fe_3_O_4_. Briefly, 8.1 g FeCl_3_·6H_2_O was dissolved in deionized water in a 250 ml conical flask and stirred with a magnetic stirrer. While slowly heating the mixture to 70°C, 4.4 g FeCl_2_·4H_2_O dissolved in deionized water was poured into the conical flask. The reaction solution was constantly stirred and its volume was maintained at 150 ml in this process. The solution was rapidly filtered with a 0.45-μm pore membrane, and 5% aqueous ammonia (NH_3_·H_2_O) at pH 10 was added dropwise to the acquired filtrate with stirring. Next, the solution was gradually warmed up to 85°C for 1 h. The precipitate was separated by using a magnet and then washed four times with absolute ethanol and deionized water to obtain the desired product.

**Preparation of Fe_3_O_4_-SiO_2_-NH_2_ and Fe_3_O_4_-NH_2_**

First, Fe_3_O_4_-SiO_2_ was prepared as follows: one gram of Fe_3_O_4_ was dispersed and mixed by ultrasonication in 80%ethanol-water solution (100 ml). Next, 1.6 ml 25% NH_3_·H_2_O was added to the solution, followed by 2 ml of TEOS. The solution was then stirred at 40°C for 4 h to obtain the desired product, Fe_3_O_4_-SiO_2_. Next, Fe_3_O_4_-SiO_2_ and nano-Fe_3_O_4_ were added separately to 50% ethanol-water solution (80 ml) and stirred rapidly. Each solution was mixed with 4 ml APTES and shaken at a rotation speed of 200 rpm and 40°C for 24 h. Each of the reaction products, Fe_3_O_4_-SiO_2_-NH_2_ (FSN) and Fe_3_O_4_-NH_2_ (FN), was washed three times with ethanol and then dried in a vacuum freeze-dryer.

**Preparation of Fe_3_O_4_-SiO_2_-NH_2_-Cellu-ZIF8**

Fe_3_O_4_-SiO_2_-NH_2_-Cellu-ZIF8 (FSNcZ8) was prepared as follows: one gram of FSN was added to 200 ml of an aqueous solution containing 12% urea and 7% sodium hydroxide. The solution was precooled at -20°C for more than 1 h and then 0.67 g of microcrystalline cellulose was added to the mixture which, was frozen for 1 h until the cellulose was dissolved. Cellulose-coated Fe_3_O_4_-SiO_2_-NH_2_-Cellu was obtained by washing the reaction mixture repeatedly with ethanol and deionized water. Then, 100 mg of the above product was dissolved in 20 ml of a zinc nitrate hexahydrate solution (40 mM), stirred for 20 min, then treated with 20 ml of 2-methylimidazole (160 mM) for 3 h. The desired product, FSNcZ8, was washed and dried.

**Immobilization of TpeRha**

The support solids (10 mg) were modified with glutaraldehyde and allowed to react for 2 h. Next, they were washed with deionized water and incubated in a mixture (5 ml) of 50 mM citric acid Na_2_HPO_4_ buffer (pH 4.5) and 2 U/ml (0.1 mg/ml) TpeRha for 12 h. The solution was washed with deionized water to remove unbound enzymes and other excess components, including excess glutaraldehyde solution, and then subjected to vacuum filtration. The properties of the modified solid supports were investigated using an EM-30 Plus Ultra High-Resolution Desktop Scanning Electron Microscope (Coxem, Korea), Fourier transform infrared spectroscopy (FTIR, Bruker, Germany) and X-ray diffraction (XRD, Bruker, Germany).

**Determination of Enzyme Activity**

The activity of free and immobilized TpeRha enzyme was measured. A mixture (100 μl) containing 1 mM p-nitrophenyl-a-L-rhamnopyranoside (*p*NPR), citric acid Na_2_HPO_4_ buffer (50mM, pH 4.5), and free or immobilized TpeRha at an appropriate dilution was incubated at 90°C in a shaking water bath for 5 min. The reaction was terminated by adding 300 μl of 1 M Na_2_CO_3_, and then absorbance at 405 nm was measured by a microplate reader (SpectraMax190). The activities of the free and immobilized enzymes, defined as a unit of enzyme activity [U], were determined by detecting the release of 1 μmol p-nitrophenyl (*p*NP) by 1 mM pNPR.

Activity recovery (%) was calculated as follows:

$$100\times \frac{\mathrm{Activity}\mathrm{of}\mathrm{immobilized}\mathrm{enzyme}}{\mathrm{Activity}\mathrm{of}\mathrm{free}\mathrm{enzymes}\mathrm{used}\mathrm{for}\mathrm{immobilization}}$$

**Determination of Protein Concentration**

The protein concentration was examined by using the Bradford Protein Assay Kit (Sangon Biotech, China) [23], and the reaction mixture contained 200 μl Bradford protein Assay Kit and 6 μl detected sample.

Protein adsorption rate (%) was calculated as follows:

$$100\times \frac{\mathrm{Protein}\mathrm{content}\mathrm{of}\mathrm{free}\mathrm{enzyme–Protein}\mathrm{content}\mathrm{in}\mathrm{supernatant}(\mathrm{mg})}{\mathrm{Protein}\mathrm{content}\mathrm{of}\mathrm{free}\mathrm{enzyme}(\mathrm{mg})}$$

**Properties of Free and Immobilized TpeRha**

The optimum temperature for the activity of the immobilized and free enzymes was investigated. The mixture (100 μl) was measured at the following temperatures: 65, 70, 75, 80, 85, 90, and 95°C. The optimum pH of the immobilized enzymes (10 mg) was tested by the pH gradient method (pH 3.0, 3.5, 4.0, 4.5, 5.0, 5.5, 6.0, 6.5, 7.0, and 8.0) at 90°C. The temperature and pH stability of the immobilized and free enzymes were observed. The immobilized and free enzymes were incubated at 65, 75, 80, and 85°C in a water bath for 3 h, and then the thermal stability of the immobilized and free enzymes was examined. The pH stability of the immobilized and free enzymes was investigated after incubation in 100 mM citric acid Na_2_HPO_4_ buffer (pH 3.5, 4.0, 4.5, 5.0, 5.5, 6.0, 6.5, 7.0, and 8.0) for 3 h at 70°C. In addition, the pH stability of the immobilized and free enzymes at high temperature was detected.

The effects of metal cations in the reaction mixture on the catalytic activities of the immobilized enzymes were determined. The metal cations tested included Fe^2+^, Li^2+^, Al^3+^, K^+^, Ni^2+^, Mn^2+^, Ca^2+^, Zn^2+^, Mg^2+^, Pb^2+^ and Cu^2+^. The effects of organic solvents, including methanol, ethanol and DMSO, were investigated. In the experiments, organic solvents at various concentrations were added to the reaction mixture to elucidate the effects of organic solvents. The effects of rhamnose and glucose concentrations on the immobilized enzymes were studied. In these reactions, the initial concentration range of rhamnose was 5-25 mM, the initial concentration range of glucose was 10-300 mM, and the temperature and pH were 90°C and 4.5, respectively.

**Application of Immobilized Enzyme in the Production of Prunin from Naringin**

A mixture (500 μl) containing 1 mmol/l naringin , citric acid Na_2_HPO_4_ buffer (50 mM, pH 5.5), and FSNcZ8-TpeRha at an appropriate dilution was incubated at 80°C in a shaking water bath for 30 min. At the end of the reaction, the final concentration of 60% ethanol was used to elute the reaction system, and a proper amount of eluate was collected and passed through the 0.22 μM filter membrane for high-performance liquid chromatography (HPLC) detection.

In this study, the effects of temperature, pH, enzyme dosage and reaction time on the transformation rate of naringin were investigated. The mixture (500 μl) was incubated at the following temperatures: 65, 70, 75, 80, and 85°C. The optimum pH was tested by the pH gradient method (pH 4.0, 4.5, 5.0, 5.5, 6.0, 6.5, and 7.0) at 80°C. The mixture (500 μl) was added at the following enzyme dosage: 0.5, 1.0, 2.0, 4.0, 6.0, 8.0, 10.0, and 12.0 U/ml at the optimum temperature and pH for 30 min. The mixture (500 μl) was reacted at the following times: 5, 10, 15, 20, 25, 30, 35, and 40 min at the optimum temperature and pH. According to the results of HPLC analysis, the optimal transformation conditions were determined. At the same time, under the condition of the same temperature, pH, enzyme dosage and reaction time, the FSNcZ8-TpeRha and free TpeRha were used for the conversion reaction respectively, and the catalytic effects were compared according to the conversion rate of naringin.

**Reusability of FSNcZ8-TpeRha**

A mixture (500 μl) containing 1 mmol/l naringin , citric acid Na_2_HPO_4_ buffer (50 mM, pH 5.5), and 6 U/mL FSNcZ8-TpeRha was incubated at 80°C in a shaking water bath for 30 min. After the reaction, the FSNcZ8-TpeRha was recovered with a magnet, washed with buffer solution for 2-3 times, and then the reaction was continued for several times with the first conversion rate of 100%, to determine its reusability.

**Determination of Naringin by HPLC**

All the transformation samples were analyzed by an HPLC 1200 system (Agilent, USA) with a C18 column (4.6 × 250 mm; i.d., 5 μm; Ser. No. USNH017518, USA). The water mobile phase was 0.1% formic acid water (solvent A) and the organic phase was methanol (solvent B). Under column temperature condition of 40°C, the substrate and product were separated by eluting for 30 min with a constant ratio of A:B = 68:32. The flow rate was 1 ml/min, and the absorbance at 269 nm was monitored.

## Results and Discussion

### Effect of Chemical Modification on FSNcZ8-TpeRha

**Fig. 1:**
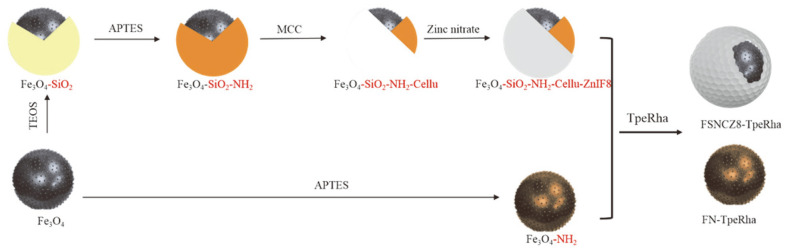
Synthesis of the magnetic microspheres Fe_3_O_4_-NH_2_, and FSNcZ8.

**Fig. 2:**
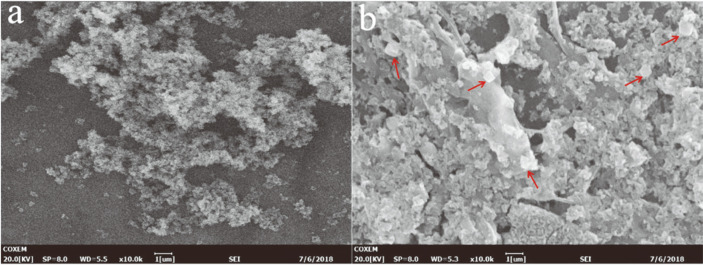
Images of modified Fe_3_O_4_ magnetic materials. (**a**) Fe_3_O_4_-NH_2_, (**b**) FSNcZ8 particles.

**Fig. 3:**
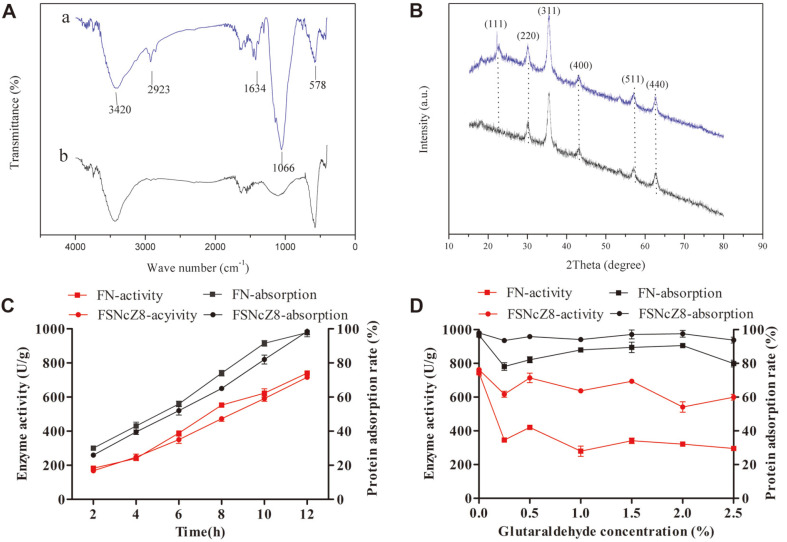
Determination of the characteristics of magnetic materials FN and FSNcZ8, and immobilization of TpeRha. **A**: Structural analysis of FN and FSNcZ8 by FITR (IR KBr (cm^−1^): 3420 (amino, N-H), 2923 (aromatic, CeH), 1634 (aromatic frame), 578 (Fe_3_O_4_) and 1066 (SiO_2_). **B**: XRD results for FNCZ8 and FSNCZ8. FN (Fig. 3B-b) produced five distinct peaks at 2θ = 30.25°, 35.56°, 43.53°, 57.82°, and 63.20°, which corresponded to the (220), (311), (400), (511), and (440) crystal planes of Fe_3_O_4_. It was shown that the amino modification has no effect on the diffraction peak of Fe_3_O_4_. FSNcZ8 (Fig. 3B-a) showed three characteristic peaks at 2θ = 20.2° ,18° and 24°, which indicated microcrystalline cellulose on the (a) crystal plane, the characteristic peaks of ZIF-8, and formation of amorphous silica, respectively.

The functional structure of the magnetic adsorption materials was activated by an amino group, and the new magnetic particles were strengthened and encapsulated with microcrystalline cellulose and sodium hydroxide (Fig. 1). Because the metal-organic framework ZIF-8 has high stability in protein-denaturing solvents and high temperature [24, 25], FSNc is coated with zinc nitrate hexahydrate and ZIF-8 framework, which provide a protective shell [26, 27]. Images indicated that the microspheres of FN (Fig. 2a) were spherical in shape with mean diameters of 220 nm, whereas the FSNcZ8 particles were cubes with mean diameters of 540 nm. This result is consistent with that of Cao Shilin *et al*. In addition, the FTIR in Fig. 3A shows an intensity peak at 1,066 cm-1 that is attributed to the SiO_2_ network. The XRD diffraction peak at 2θ =24° (Fig. 3B) indicates the formation of amorphous silica and two characteristic peaks at 2θ = 20.2° and 18°, which were the diffraction peaks of microcrystalline cellulose on the (a) crystal plane and the characteristic peaks of ZIF-8 [17]. Both methods demonstrate the effectiveness of the synthesis of FSNcZ8. Both FN and FSNCZ8 were used to adsorb TpeRha. The results are shown in Figs. 3C and 3D. As presented in Fig. 3C, the highest adsorption rate, enzyme activity and enzyme activity recovery rate obtained with FSNcZ8 as support were 98.44% (49.22 mg protein/1 g FSNcZ8), and 716 U/g and 72.53% after 12 h of reaction, respectively, which was similar to FN (97.81%, 740 U/g, 75.65%).

To increase TpeRha loading and decrease enzyme loss, glutaraldehyde was added to provide crosslinked polystyrene, which enabled covalent coupling of the free amino groups of TpeRha and the free aldehyde group of glutaraldehyde [28]. The glutaraldehyde molecule is significantly large, which allows the formation of a spacer arm with multiple groups that can interact with the enzyme in various ways depending on the immobilization conditions [29, 30]. The results are shown in Fig. 3D. Glutaraldehyde significantly affected the adsorption and activity of the immobilized enzymes. As glutaraldehyde concentration increased, the activity of the immobilized enzyme gradually decreased. FN and FSNcZ8 containing 0.5% glutaraldehyde achieved the highest enzyme loading, whereas the highest activities of FN-TpeRha and FSNcZ8-TpeRha approximated 420 U/g and 714 U/g, respectively.

### Properties of FN-TpeRha and FSNcZ8-TpeRha

**Fig. 4:**
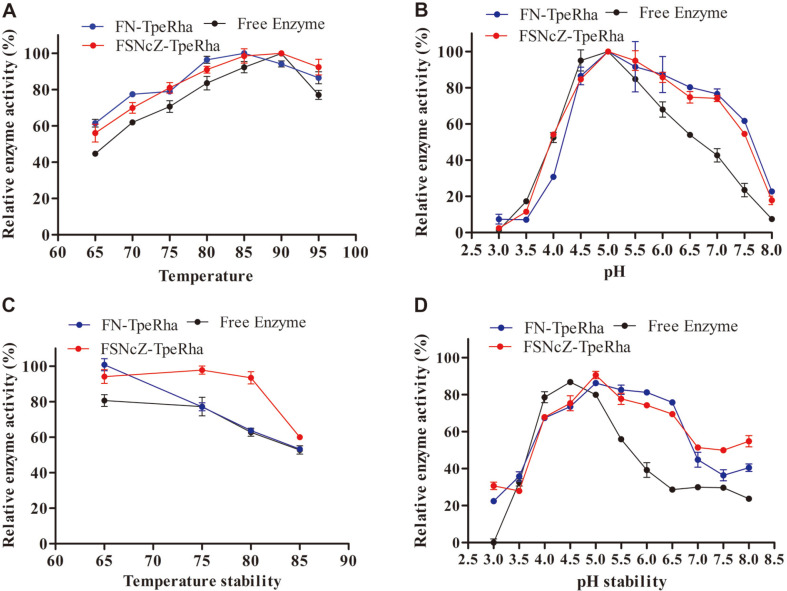
Properties of Free Enzyme, FN-TpeRha and FSNcZ8-TpeRha. **A**, Optimum temperature; **B**, optimum pH; **C**, temperature stability; **D**, pH stability. The initial activity at the optimum condition was defined as 100 %, and these activities are expressed as relative values.

The optimum temperature and pH of FN-TpeRha and FSNcZ8-TpeRha were measured, and the temperature was set between 65-95°C to examine the high-temperature properties of immobilized TpeRha. The pH was increased from 3.0 to 8.0. The results are presented in Figs. 4A and 4B. The optimum temperature and pH of FN-TpeRha were 85°C and 5.0, respectively. The optimum reaction conditions of FSNcZ8-TpeRha were 90°C and pH 5.0. On the other hand, the activities of the immobilized enzymes were more maintained than that of the free enzyme. Notably, the free enzyme was fully inactivated under relatively extreme acid or alkali conditions.

The effect of temperature variations on the activities of free and immobilized TpeRha was measured. The magnetic microspheres were incubated at different temperatures (65, 75, 80, or 85°C) for 3 h. Fig. 4C clearly shows that the high-temperature stability of the immobilized enzymes, especially FSNcZ8-TpeRha, was higher than that of the free enzyme. After incubation at 80oC for 3 h, the residual enzyme activities of FSNcZ8-TpeRha, FN-TpeRha and free enzyme were 93.5%, 63.32% and 62.77%, respectively. The enzyme-MOF composites have shown great potential in increasing enzyme stability at high temperature. The results also showed that the immobilized enzyme with a cellulose coating had good thermal stability, which was consistent with the results of Wu R *et al*.[31]. The thermal stability of TpeRha on FN-TpeRha and FSNcZ8-TpeRha preparations is one of the most important criteria for use in different applications. The pH stabilities of the magnetic microspheres are presented in Fig. 4D (pH 3.5, 4.0, 4.5, 5.0, 5.5, 6.0, 6.5, 7.0, and 8.0). The free enzyme may experience protein aggregation (mainly near to the isoelectric point). This may be caused by undesired enzyme interactions where inactivation can stabilize incorrect enzyme structures. Under alkaline conditions, the immobilized enzymes maintained higher activity than that of the free enzyme; however, the instability of the magnetic materials under acidic conditions might lead to low enzyme activity. The results showed that FSNcZ8 was better at immobilizing TpeRha and it could maintain the spatial structure and saturation of enzyme molecules, as well as resist the interference of high temperature and extreme pH.

### Effect of Metal Ions on the Activity of FSNcZ8-TpeRha

The effects of various metal cations on the catalytic efficiency of immobilized enzymes have not been studied thoroughly [32-34]. Based on known interactions between free enzymes and metal ions, the effects of metal cations on the activities of FSNcZ8-TpeRha and free TpeRha were determined at pH 5.0 and at 90°C. The final concentrations of metal cations in the reaction solutions were 2 mM and 5 mM. The results presented in Table 1 show that the activity of free TpeRha is inhibited by Cu^2+^ Zn^2+^ and Mn^2+^ at the concentration of 5 mM, showing that the immobilized enzyme can effectively alleviate the inhibition of these ions. Also, free TpeRha is activated by K^+^ and Li^+^ at the concentration of 2 mM. Meanwhile, the effect of metal ions on FSNcZ8-TpeRha is similar to that of free enzyme.

### Effects of Organic Solvents on the Activity of Enzyme

In reaction systems involving biological enzymes, organic solvents and metal ions are often involved. These factors greatly influence the industrial catalytic conversion of bioenzymes; they affect the catalytic ability and specificity of the enzymes. Under ethanol, methanol, and DMSO concentrations of 15%, the residual activities of free TpeRha were more than 29.8% , 43.9%, and 41.0% of the initial activity, respectively (Table 2), whereas those of FSNcZ8-TpeRha were 53.6%, 61.4%, and 53.9%, respectively. The results showed that FSNcZ8 was a potential carrier of TpeRha, which can improve the enzymatic properties of organic solvents used in the reaction system.

### Monosaccharide Tolerance of TpeRha and FSNcZ8-TpeRha

**Fig. 5:**
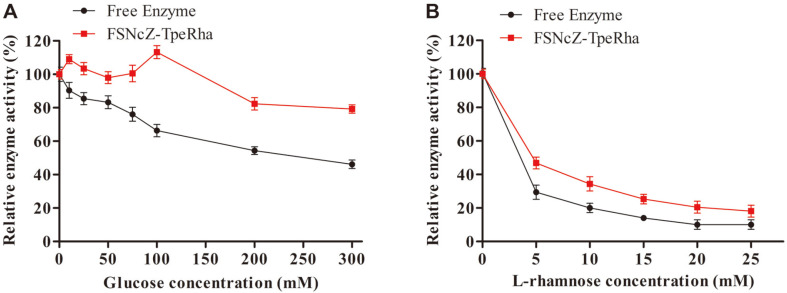
Monosaccharide tolerance of Free Enzyme and FSNcZ8-TpeRha. **A**, Glucose; **B**, L-rhamnose.

Numerous monosaccharides are generated and accumulated following glycoside hydrolysis. However, under high monosaccharide concentration, the substrate affinity of the enzyme toward monosaccharides may increase, thus inhibiting glycoside hydrolysis and leading to decreased output of the desired product. In addition, the enzymatic hydrolysis reaction involved with rhamnosidases is often accompanied by the degradation of glucosidic bonds by glucosidase. For rhamnosidase to efficiently hydrolyze a glycoside compound containing L-rhamnose, it is extremely important to investigate the tolerance of rhamnosidase to glucose and L-rhamnose [35], and the results are shown in Figs. 5A and 5B. The catalytic activity of free TpeRha decreased rapidly as glucose concentration increased to 300 mM, and only 22% of the initial activity was retained at 200 mM glucose. The catalytic activity of free TpeRha decreased rapidly as glucose concentration increased to 300 mM, and only 22% of the initial activity was retained at 200 mM glucose. However, the activity of FSNcZ8-TpeRha was maintained at 48% of the initial activity, which may indicate that the structure of the magnetic microsphere protected the enzyme active site, thus decreasing the influence of high glucose concentration.

However, the rhamnose tolerance of TpeRha (Fig. 5B), unlike its inhibition at high-glucose conditions, was significantly inhibited by L-rhamnose at concentrations of below 10 mM. Moreover, the residual activities of FSNcZ8-TpeRha were maintained at 47% of the initial activity, which was 18% higher than that of free TpeRha at 5 mM rhamnose. The results showed that the structure of FSNcZ8 led to a reduction in direct interaction between the enzyme and L-rhamnose to a certain extent, protecting the activity of the enzyme. On the other hand, it was indicated that high-temperature rhamnosidase was highly susceptible to direct inhibition by L-rhamnose.

### Application of FSNcZ8-TpeRha

**Fig. 6:**
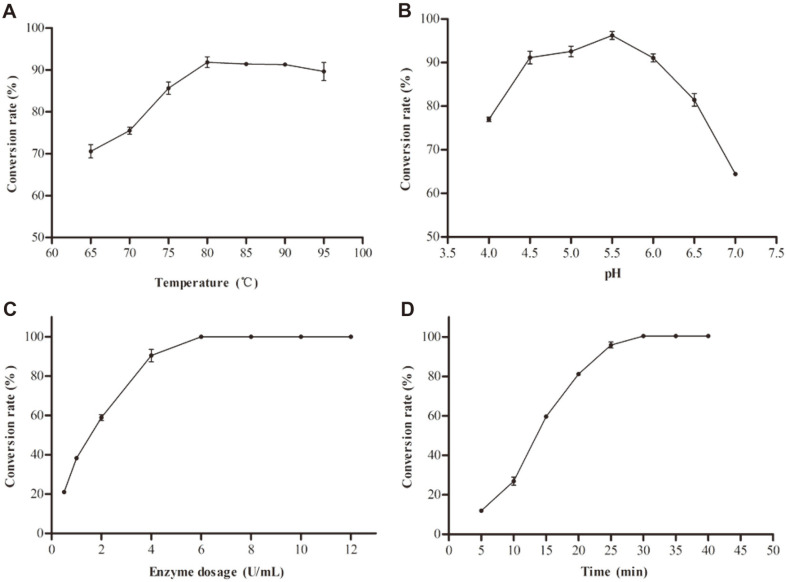
The optimum conditions for the transformation of naringin catalyzed by FSNcZ8-TpeRha. **A**, Optimum temperature; **B**, optimum pH; **C**, enzyme dosage; **D**, reaction time.

**Effect of temperature and pH on transformation.** HPLC of naringin to prunin catalyzed by immobilized enzyme is shown in Fig. S1. When FSNcZ8-TpeRha catalyzes the transformation of naringin, the conversion rate increases gradually at 65-80°C with the increase of temperature (as shown in Fig. 6A). The reaction reached equilibrium at 80-90°C, and the catalytic effect of FSNcZ8-TpeRha was the same at this temperature, but the conversion rate decreased at 95°C, which may be caused by the decrease of enzyme activity at 95°C. According to the temperature and pH stability test results of FSNcZ8-TpeRha, 80°C was selected as the optimal temperature for conversion, and pH 5.5 as the optimal pH for conversion.

**The effect of enzyme dosage on the transformation.** The effect of enzyme dosage on the conversion rate is shown in Fig. 6C. The conversion rate increased with the increase of enzyme dosage, and reached 100% when the enzyme amount was 6-12 U/ml. Therefore, 6 U/ml was selected as the optimal enzyme dosage.

**The effect of reaction time on the transformation.** The final concentration of 6 U/ml FSNcZ8-TpeRha was used to transform 1 mmol/l naringin, and the change of transformation with time was shown in Fig. 6D. With the reaction going on, the conversion rate of naringin increased, and the conversion rate reached 100% at 30 min.

### Comparison of Catalytic Performance

**Fig. 7:**
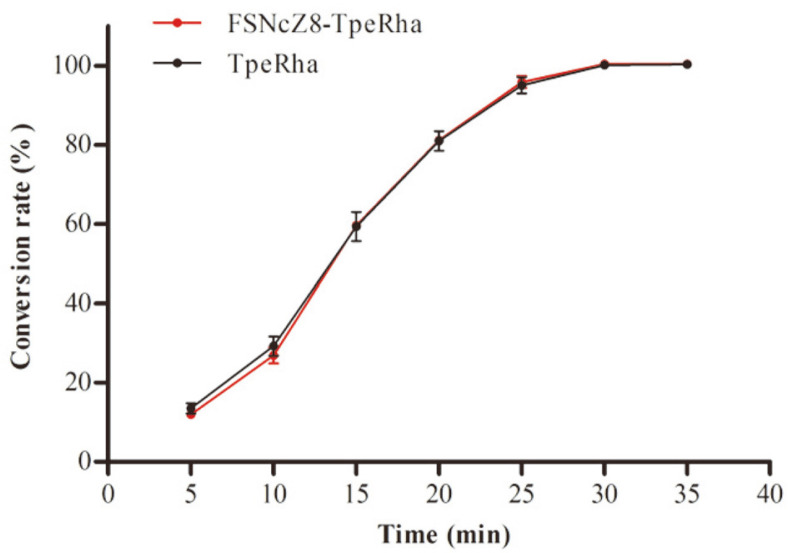
Comparison of catalytic effects of TpeRha and FSNcZ8-TpeRha.

Under the same conditions, free TpeRha and FSNcZ8-TpeRha were used to catalyze the transformation of 1 mmol/l naringin. The results are shown in Fig. 7. The conversion rate of naringin increased with time. In the same reaction time (85°C, pH 5.5, enzyme dosage of 6 U/ml), the conversion of naringin catalyzed by FSNcZ8-TpeRha was the same as that of free TpeRha. After the reaction for 30 min, the conversion rates of both can reach 100%, indicating that the catalytic activity of the immobilized enzyme prepared by this method is not restricted by the carrier and can reach the catalytic level of free enzyme under certain conditions.

### Reusability of FSNcZ8-TpeRha

**Fig. 8:**
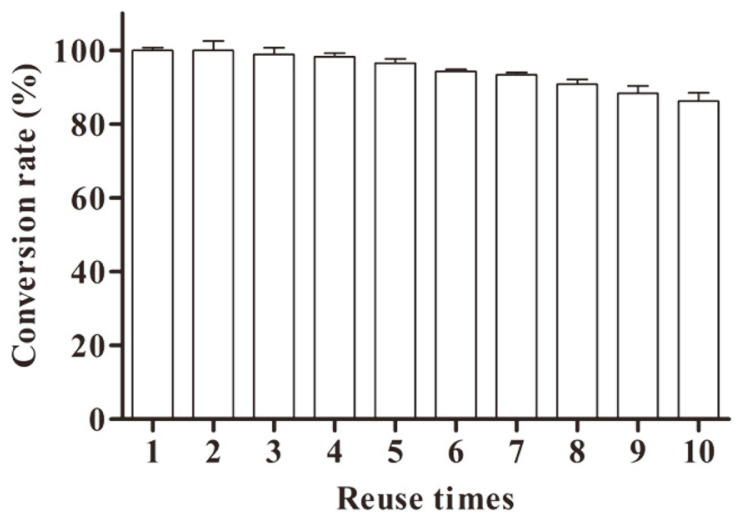
Reusability of FSNcZ8-TpeRha.

The non-reusability of free enzymes is one of the most important factors that limit their application in industry. It is an important way to reduce the cost of enzyme by using immobilized enzyme technology to improve the reuse rate. As presented in Fig. 8, the conversion rate of naringin remained above 80% after repeating the reaction of FSNcZ8-TpeRha 10 times, which was conducive to the industrial application of thermophilic α-L-rhamnosidase.

## Supplemental Materials



Supplementary data for this paper are available on-line only at http://jmb.or.kr.

## Figures and Tables

**Table 1:** Effects of the metal cations on the free TpeRha and FSNcZ-TpeRha.

Metal cation	Free TpeRha		FSNcZ8- TpeRha	

	2mM	5mM	2mM	5mM
Zn^2+^	91.52±2.2	69.37±7.1	87.24±4.2	82.43±12
Ba^2+^	96.97±6.0	93.23±2.5	89.91±1.7	87.89±0.2
K^+^	105.45±2.8	105.38±2.7	98.85±3.4	90.23±2.5
Mg^2+^	100.80±2.1	91.21±5.2	95.14±2.5	86.25±4.0
Mn^2+^	102.48±4.4	86.79±0.4	102.55±7.5	107.36±2.6
Ca^2+^	102.97±7.5	87.48±0.7	89.61±5.6	85.22±3.1
Al^3+^	102.66±1.9	88.65±0.2	91.43±2.6	88.22±3.9
Li^+^	105.26±3.3	89.66 ±4.2	98.06±9.6	94.60±11
Pb^2+^	102.48±3.7	99.01±0.8	103.50±1.6	108.10±0.9
Cu^2+^	63.00±2.2	45.00±2.2	88.61±1.2	75.45±0.4

**Table 2:** Effects of the organic solvents on the free TpeRha and FSNcZ-TpeRha.

Organic solvents（15%）	Free TpeRha	FSNcZ8-TpeRha
Ethanol	29.8 ±5.2	53.6 ±0.3
Methanol	43.9 ±0.3	61.4 ±2.3
DMSO	41.0 ±2.7	53.9 ±1.8
