# Supplementary figures and images for: Immobilization of GH78 ⓹-L-Rhamnosidase from Thermotoga petrophilea with High-Temperature-Resistant Magnetic Particles Fe3O4-SiO2-NH2-Cellu-ZIF8 and Its Application in the Production of Prunin Form Naringin
Source: J Microbiol Biotechnol. 2020 Jun 24;31(3):419–28. doi: 10.4014/jmb.2004.04055 (PMC9705872; doi:10.4014/jmb.2004.04055)

## S1. HPLC of naringin and prunin

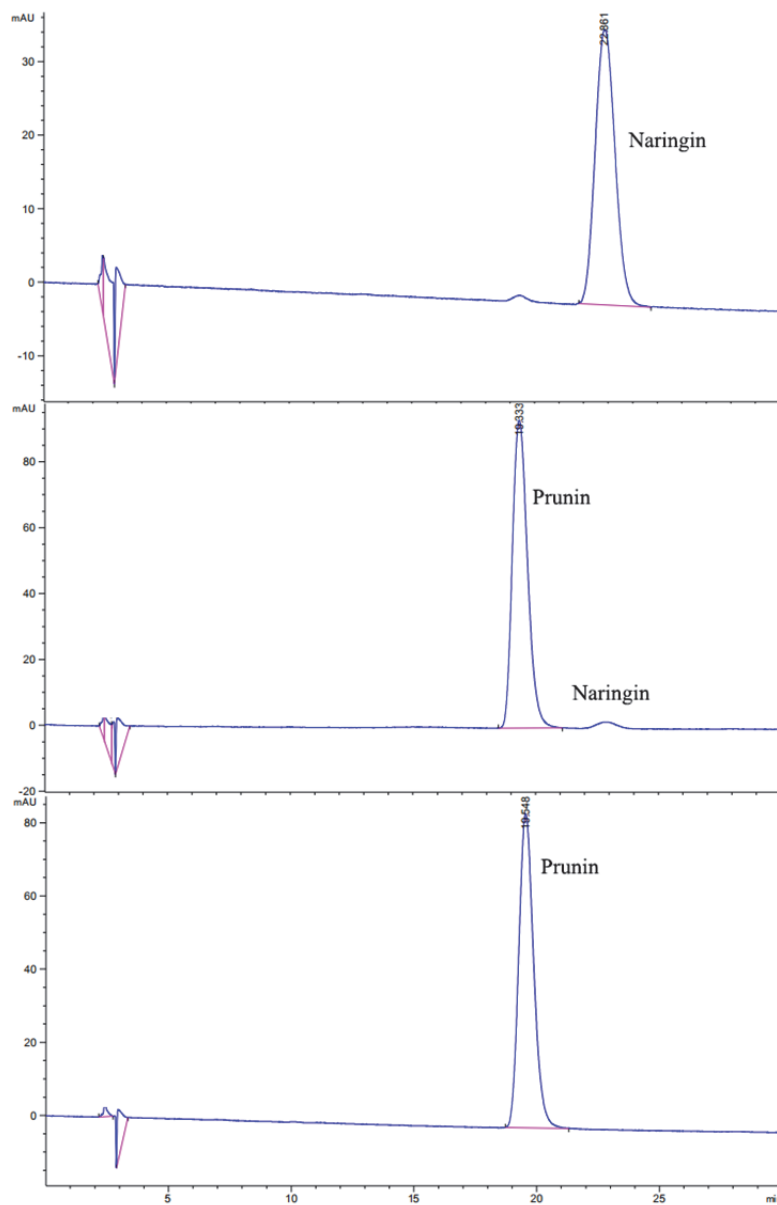

Supplement: Supplementary file 1 [file jmb-31-3-419-supple.pdf]
